# Differences in pregnancy outcomes and characteristics between insulin- and diet-treated women with gestational diabetes

**DOI:** 10.1186/s12884-015-0706-x

**Published:** 2015-10-23

**Authors:** Katrien Benhalima, Karolien Robyns, Paul Van Crombrugge, Natascha Deprez, Bruno Seynhave, Roland Devlieger, Johan Verhaeghe, Chantal Mathieu, Frank Nobels

**Affiliations:** Department of Endocrinology, UZ Gasthuisberg, KU Leuven, Herestraat 49, 3000 Leuven, Belgium; Department of Endocrinology, OLV ziekenhuis Aalst-Asse-Ninove, Moorselbaan 164, 9300 Aalst, Belgium; Department of Obstetrics and Gynecology, OLV ziekenhuis Aalst-Asse-Ninove, Moorselbaan 164, 9300 Aalst, Belgium; Department of Obstetrics and Gynecology, UZ Gasthuisberg, KU Leuven, Herestraat 49, 3000 Leuven, Belgium

**Keywords:** Gestational diabetes, Treatment, Insulin, Diet

## Abstract

**Background:**

Our aim was to evaluate the difference in pregnancy outcomes and characteristics between insulin- and diet-treated women with gestational diabetes (GDM).

**Methods:**

Retrospective analysis of the medical files from 2010–2013 of women with GDM diagnosed with the Carpenter & Coustan criteria attending two clinics, one in a university and another in a non-university hospital. Characteristics associated with insulin use were analyzed. Multivariable logistic regression was used to adjust for confounders. For women attending the university hospital, indices of insulin sensitivity such as the reciprocal of the homeostasis model assessment of insulin resistance (1/HOMA-IR) and an index of beta-cell function, the Insulin Secretion-Sensitivity Index-2 (ISSI-2) were calculated.

**Results:**

Over a 4 year period, 601 women were identified with GDM of whom 22.9 % were obese at first prenatal visit. 24.2 % needed insulin. Insulin did not prevent adverse outcomes, as women on insulin had higher rates of large-for-gestational age infants (LGA) (28.5 % vs. 13.1 %, *p* < 0.0001) and more cesarean sections (44.1 % vs. 27.0 %, *p* = 0.001), remaining significant after adjustment for confounders. Compared to diet-treated women, women on insulin more often had an ethnic minority background (33.3 % vs. 21.6 %, *p* = 0.004), more often had a history of GDM (21.5 % vs. 10.4 %, *p* = 0.002), were more often multiparous (59.3 % vs. 47.6 %, *p* = 0.044) and were diagnosed with GDM earlier in pregnancy (weeks 25.3 ± 4.9 vs. 27.1 ± 3.7, *p* < 0.0001). When undergoing an oral glucose tolerance test, women treated with insulin had a higher fasting glycaemia (97.6 ± 18.8 vs.87.7 ± 10.3, *p* < 0.0001), a higher 1-hour glycaemia (197.7 ± 30.1 vs.184.5 ± 25.8, *p* < 0.0001), a higher 2-hour glycaemia (185.2 ± 28.5 vs. 175.0 ± 22.8, *p* < 0.0001), more often 3 and 4 abnormal values (58.1 % vs. 37.8 %, *p* < 0.0001 and 24.8 % vs. 7.7 %, *p* < 0.0001) and higher HbA1c levels (5.5 ± 0.6 vs 5.2 ± 0.5, *p* < 0.0001). ISSI-2 (1.3 ± 0.5 vs. 1.7 ± 0.5, *p* < 0.0001) and 1/HOMA-IR [0.01 (0.001–0.002) vs. 0.02 (0.01–0.03), *p* = 0.027] were lower in women on insulin. Women on insulin more often received corticoids in preparation of preterm delivery (11.0 % vs. 2.4 %, *p* < 0.0001).

**Conclusion:**

Compared to diet-treated women with GDM, women treated with insulin have a higher risk profile, impaired beta-cell function and lower insulin sensitivity. Rates of LGA and cesarean sections were higher in insulin-treated women.

## Background

Gestational diabetes (GDM) is a frequent medical condition during pregnancy and was historically defined as ‘any degree of glucose intolerance with onset or first recognition during pregnancy’ [1]. GDM has long been known to raise the risk of a large-for-gestational age infant (LGA) and macrosomia resulting in increased rates of shoulder dystocia and caesarian deliveries [2, 3]. Shortly after the delivery the glucose values are generally restored to normal, but women with GDM have a seven-fold increased risk of developing type 2 diabetes (T2DM) [4]. Two large randomized intervention trials have demonstrated improvement in perinatal outcomes in the group of women who received treatment of mild GDM, especially in the frequency of LGA and preeclampsia [5, 6]. Initial treatment of GDM involves diet modification, glucose monitoring and moderate exercise [7]. If lifestyle is insufficient to maintain the glycemic targets, pharmacological therapy becomes necessary. Insulin has been the treatment of choice when lifestyle measures do not maintain glycemic control during pregnancy. Recent studies have suggested that metformin and glibenclamide (glyburide) may be safe and acceptable alternatives but there is a paucity of long-term follow up data on children exposed to oral agents in utero [8, 9]. Recent data also point towards the fact that sulfonylurea do cross the placenta when used in high doses [10]. The American Diabetes Association (ADA) therefore only recommends insulin as pharmacotherapy for GDM [7]. The difference in pregnancy outcomes between insulin- and diet-treated women with GDM and the profile of women where insulin-therapy is warranted, remain unclear. The aim of our study was therefore to evaluate the pregnancy outcomes and characteristics in insulin- versus diet-treated women with GDM in Belgian patients.

## Methods

Retrospective analysis of the electronic medical files of all women with GDM diagnosed with the Carpenter & Coustan criteria attending two large obstetrical centers in Belgium (the University Hospital UZ Leuven and the non-university hospital OLV Aalst-Asse-Ninove) from 01–01–2010 till 31–12–2013. The study was approved by the Institutional Review Board of UZ Leuven (ML 10085) and by the Institutional Review Board of the hospital OLV Aalst-Asse-Ninove (B126201422577). Due to the retrospective nature of the study there was no need for informed consent from the participants as in compliance with the Belgian Law of December 8,1992 on the protection of privacy and the Belgian Law of August 22, 2002 on the rights of the patient.

Approximately 2400 women are delivered annually at UZ Leuven and about 1700 women at the hospital OLV Aalst-Asse-Ninove. The background prevalence of T2DM in the adult Belgian population is 6.5 % [11]. In the general adult population 28 % of women are overweight and 13 % are obese [12]. Accurate data on the prevalence of GDM are lacking in Belgium and the current practice for screening for GDM varies across different centers [13]. In both centers, women were not yet universally screened for overt diabetes early in pregnancy. All pregnant women were screened and diagnosed for GDM according to the Fifth International Workshop Conference criteria [14]. Women received a 50 g glucose challenge test (GCT) between 24–28 weeks and those testing positive [threshold after 1-h ≥ 140 mg/dl (7.8 mmol/l)] had a 3-hour 100 g oral glucose tolerance test (OGTT) within two weeks after the GCT using the Carpenter & Coustan criteria for GDM [fasting plasma glucose (FPG) ≥ 95 mg/dl (5.3 mmol/l), 1-hour glycaemia ≥ 180 mg/dl (10.0 mmol/l), 2-hour glycaemia ≥ 155 mg/dl (8.6 mmol/l) and 3-hour glycaemia ≥ 140 mg/dl (7.8 mmol/l), diagnosis of GDM if ≥ 2 values are abnormal]. Although there are no specific recommendations in both centers on which women should receive screening for GDM before 24 weeks of pregnancy, screening for GDM with a 50 g GCT is sometimes performed before 24 weeks of pregnancy in high risk women such as women with a history of GDM. Women with GDM were treated with insulin when despite lifestyle measures the FPG was ≥ 95 mg/dl (5.3 mmol/l) and/or 2-hour postprandial glycaemia ≥120 mg/dl (6.7 mmol/l). To uniform the initiation for insulin therapy as much as possible, the ‘Weekly Average Glycaemia’ (WAG) is calculated based on the self-monitoring values of the blood glucose (fasting and postprandial) during the first weeks after the diagnosis. Therapy with insulin is initiated when the fasting WAG and/or the postprandial WAG is above target during two weeks in a row. The treatment and follow up policies were identical in both centers. In both centers oral anti-diabetes drugs such as metformin and glibenclamide (glyburide) are not routinely used during pregnancy.

Outcomes were obtained from review of the electronic database. Maternal characteristics recorded were age, ethnicity, weight, body mass index (BMI) at first prenatal visit and at delivery, overweight (BMI ≥25 Kg/m^2^), obesity (BMI ≥ 30 Kg/m^2^), weight gain (difference in weight between first prenatal visit and the delivery), parity, a history of a first degree or second degree relative with diabetes and history of GDM. Excessive weight gain was defined according to the most recent Institute of Medicine (IOM) guidelines [15]. Other data that were recorded are: the glucose values and the insulin values (only for UZ Leuven) based on the 100 g OGTT during pregnancy (0 min-60 min-120 min-180 min), gestational age at delivery, the timing and result of the GCT, the gestational age at the diagnosis of GDM, HbA1c at the time of the 100 g OGTT during pregnancy, whether women received treatment with corticoids during pregnancy, need of insulin, type of insulin and number of injections and the gestational age at the start of insulin.

The following maternal pregnancy outcomes were recorded: gestational hypertension (blood pressure ≥ 140/90 mmHg), preeclampsia [hypertension with proteinuria or in combination with reduced fetal growth or the ‘Hemolysis Elevated Liver enzymes and Low Platelets’ (HELPP)-syndrome], preterm delivery (<37 weeks of gestation) and cesarean section (planned + emergency sections combined). The following neonatal outcomes were recorded: birth weight, macrosomia (birth weight > 4 kg), large-for-gestational age infants (LGA, birth weight >90 percentile adjusted for sex and parity), small-for-gestational age infants (SGA, birth weight <10 percentile adjusted for sex and parity), shoulder dystocia, Apgar score (at five minutes) and admission at the neonatal intensive care unit (NICU).

Since insulin levels were not available from women from the OLV Aalst-Asse-Ninove hospital, indices of insulin sensitivity and an index of beta-cell function were only analyzed from women attending the university hospital UZ Leuven. Insulin sensitivity was measured using the insulin sensitivity index of Matsuda, a well-established measure of whole-body insulin sensitivity [16]. The insulin sensitivity index of Matsuda is defined as 10 000/√ [(FPG x fasting plasma insulin) X (mean glucose during OGTT x mean insulin during OGTT)] [16]. As a secondary measure of insulin sensitivity (largely hepatic), we also calculated the reciprocal of the homeostasis model assessment of insulin resistance (1/HOMA-IR) [17]. HOMA-IR is calculated as the product of FPG and fasting plasma insulin divided by 22.5 [17]. Beta-cell function was assessed by the insulin secretion sensitivity index (ISSI-2), an OGTT-derived measure that is analogous to the disposition index obtained from the frequently sampled intravenous glucose tolerance test [18, 19]. Glycaemia was assessed by the area under the glucose curve (AUC glucose) during the OGTT, calculated using the trapezoidal rule [18, 19]. ISSI-2 is defined as the product of 1) insulin secretion measured by the ratio of the AUC insulin to the AUC glucose and 2) insulin sensitivity measured by the insulin sensitivity index of Matsuda [18, 19]. All these measures have been validated for use in pregnancy.

In UZ Leuven, HbA_1c_ was measured by reversed-phase cation-exchange chromatography (ADAMS HA-8160, Menarini Diagnostics Benelux, Zaventem, Belgium) and in OLV Aalst-Asse-Ninove hospital with a capillary zone electrophoresis on a Capillarys 2 Flex Piercing (Sebia). Hba1c is reported in compliance with the National Glycohemoglobin Standardization Program (NGSP). In Both centers plasma glucose was measured by an automated colorimetric-enzymatic method (hexokinase-glucose-6-phosphate-dehydrogenase, application 668) on a Hitachi/Roche-Modular P analyzer. Insulin was measured by the immunometric ECLIA (Roche Modular E170, Basel, Switserland).

### Statistical analyses

Statistical analyses were performed using SPSS 22.0. Continuous variables were presented as mean and standard deviation if normally distributed and as median otherwise.

Categorical variables were presented as percentage. To compare variables between two groups independent samples *t*-tests were used for normally distributed continuous variables, Mann–Whitney’s *U*-test for non-normally distributed continuous variables and chi-squared tests for categorical variables. A univariable analysis was done initially, and then clinical variables that were identified as most significantly associated with the need for insulin use were included in a multivariable logistic regression. Treatment with corticoids was a significant variable in the univariable analysis but this was not included in multivariable logistic regression since it could not be excluded that treatment with corticoids preceded the use of insulin in some patients. Multivariable logistic regression was also used to analyse the impact of possible confounders such as age, BMI, ethnicity, multi-parity, excessive weight gain and center on pregnancy outcomes and on the indices of insulin sensitivity and beta-cell function. Although the treatment protocol for GDM was similar in both centers, a center was also used as a variable to adjust for differences in population characteristics and potential differences in obstetrical management between both centers. A *p*-value of <0.05 (two-tailed) was considered significant.

## Results

Over a 4 year period, 644 women were identified with GDM. After evaluation of the medical files, 43 files were not included in the analysis due to insufficient data (29) or because women took part in a study using the ‘International Association of Diabetes and Pregnancy Study groups’ (IADPSG) criteria for GDM (14), leaving a cohort of 601 women with GDM for analysis. Of the whole cohort 269 women (44.8 %) attended the university hospital and 332 women (55.2 %) attended the non-university hospital. The prevalence of GDM at the time of the study was 3.3 % in the university center and 5.1 % in the non-university center. Of the whole cohort, 29.7 % had an abnormal fasting glycaemia on the OGTT, 64.1 % had an abnormal 1-hour value, 90.5 % had an abnormal 2-hour value and 68.7 % had an abnormal 3- hour value. In 47.4 % of women the 3-hour value on the OGTT contributed to the diagnosis of GDM. Table 1 gives an overview of the general characteristics of the cohort. Of all women with GDM 24.5 % were from an ethnic minority background (EMB): South Asian (25.3 %), Northern-African (19.8 %), Black African (18.4 %) and Middle-East (13.0 %). 24.1 % (145) of women needed insulin during pregnancy. Of all women on insulin, 69.1 % (92) received only short acting insulin, 2.3 % (3) received only long-acting insulin and 28.6 % (38) received both short -and long acting insulin. Of all women on short acting insulin, 57.1 % (76) received short acting insulin at each meal. The most commonly used short acting insulins were the insulin analogues aspart or lispo (92.9 %) and the most commonly used long acting insulin was NPH insulin (90.2 %). The insulin was stopped after the delivery except in two women. The general characteristics were pretty similar between both centers. In particular, rates of insulin use were not significantly different between the university and non-university center (27.2 % vs. 21.8 %, *p* = 0.291). Table 1 gives an overview of the similarities and differences between both centers.

**Table 1:** The general characteristics and the differences between both centers of the cohort of women with GDM

	General cohort *N* = −601	University hospital *N* = 269 (44.8 %)	Non-University hospital *N* = 332 (55.2 %)	*p*-value
Mean age (years)	31.9 ± 4.8	32.0 ± 4.8	31.5 ± 4.8	**0.028**
% first degree relative with diabetes	18.3 % (109)	14.9 % (40)	21.1 % (69)	**<0.0001**
% second degree relative with diabetes	21.8 % (130)	8.2 % (22)	33.0 % (108)	**<0.0001**
% with a history of GDM	13.1 % (78)	12.3 % (33)	13.8 % (45)	**0.023**
% overweight	32.2 % (158)	34.2 % (89)	30.0 % (69)	0.317
% obese	22.9 % (112)	22.3 % (58)	23.5 % (54)	0.758
% excessive weight gain	19.7 % (103)	22.0 % (55)	17.5 % (48)	0.197
% EMB	24.5 % (146)	37.2 % (100)	14.1 % (46)	**<0.0001**
Fasting glycaemia at OGTT in mg/dl (mmol/l)	90.0 ± 13.4 (5.0 ± 0.7)	93.6 ± 15.6 (5.5 ± 0.9)	87.3 ± 10.8 (4.8 ± 0.6)	**<0.0001**
HbA1c, mean in % (mmol/mol)	5.3 ± 0.5 (34 ± 6)	5.4 ± 0.5 (36 ± 6)	5.1 ± 0.4 (32 ± 5)	**<0.0001**
% Insulin use	24.1 % (145)	27.2 % (73)	21.8 % (72)	0.291
% macrosomia	8.7 % (52)	7.5 % (20)	9.7 % (32)	0.329
% LGA	16.9 % (101)	16.8 % (45)	17.1 % (56)	0.927
% Preterm delivery	13.0 % (187)	18.3 % (49)	8.8 % (29)	**<0.0001**
% cesarean section	31.2 % (187)	36.6 %(98)	26.96 % (89)	**0.011**
% shoulder dystocia	1.2 % (7)	2.2 % (6)	0.3 % (1)	**0.029**
% admission NICU	9.6 % (57)	16.4 % (44)	4.0 % (13)	**<0.0001**

*GDM* gestational diabetes, *EMB* ethnic minority background, *OGTT* oral glucose tolerance test, *LGA* large-for-gestational age infants, *NICU* neonatal intensive care unit, P-values in bold are significant values

Table 2 gives an overview of the differences in pregnancy outcomes of the whole cohort between the insulin- and diet-treated women GDM. Insulin did not prevent adverse outcomes, as women on insulin had higher rates of LGA (28.5 % vs. 13.1 %, *p* < 0.0001) and more cesarean sections (44.1 % vs. 27.0 %, *p* = 0.001), remaining significant after adjustment for age, BMI, excessive weight gain, ethnicity, multi-parity and center.

**Table 2:** The differences in pregnancy outcomes between the insulin- and diet-treated women with GDM

	Diet *N* = 456 (75.9 %)	Insulin *N* = 145 (24.1 %)	*p*-value	Adjusted *p*-value
% Gestational hypertension	7.7 % (35)	4.2 % (6)	0.140	
% preeclampsia	4.0 % (18)	7.6 % (11)	0.076	
% Preterm delivery	12.8 % (58)	13.9 % (20)	0.743	
% cesarean section	27.0 % (122)	44.1 % (64)	**<0.0001**	**0.001**
Birth weight (g)	3208.2 ± 600.9	3321.4 ± 589.2	**0.049**	0.144
% macrosomia	7.3 % (33)	12.6 % (18)	**0.049**	0.226
% LGA	13.1 % (59)	28.5 % (41)	**<0.0001**	**<0.0001**
% SGA	11.3 % (51)	8.3 % (12)	0.309	
% shoulder dystocia	1.1 % (5)	1.4 % (2)	0.788	
% low Apgar score	2.2 % (10)	2.8 % (4)	0.702	
% admission NICU	8.7 % (39)	12.5 % (18)	0.174	

*g* grams, *LGA* large-for-gestational age infants, *SGA* small-for-gestational age infants; low Apgar score: <7 at 5 min; *NICU* neonatal intensive care unit; The *p*-values are adjusted for age, BMI, excessive weight gain, ethnicity, multi-parity and center, P-values in bold are significant values

Table 3 gives an overview of the differences in characteristics of the whole cohort between the insulin- and diet-treated women with GDM. Compared to diet-treated women, women on insulin were more often from an EMB (33.3 % vs. 21.6 %, *p* = 0.004), more often had a history of GDM (21.5 % vs. 10.4 %, *p* = 0.002), were more often multiparous (59.3 % vs. 47.6 %, *p* = 0.044) and were diagnosed with GDM earlier in pregnancy. Women on insulin also had higher glycaemic values on the OGTT and more often 3 and 4 abnormal values with higher HbA1c levels at the time of the OGTT (Table 3). In the multivariable regression analysis fasting glycaemia at the time of the OGTT remained the only independent predictor for antenatal insulin requirement (*p* < 0.0001) (Table 3). Based on a receiver operating characteristics (ROC) curve for FPG with an AUC of 0.676, a FPG cut-of ≥ 88.5 mg/dl (4.9 mmol/l) had the best sensitivity and specificity combined of resp. 62.6 % and 60.6 % to predict insulin requirements. 62.8 % of women who had a FPG ≥ 88.5 mg/dl (4.9 mmol/l) on the OGTT required insulin treatment but with a low positive predictive value of 52.3 %.

**Table 3:** The differences in characteristics between the insulin- and diet-treated women with GDM

	Diet *N* = 456 (75.9 %)	Insulin *N* = 145 (24.1 %)	*p*-value	*Adjusted *p*-value
Age, mean (y)	31.8 ± 4.8	32.5 ± 4.7	0.109	
Gestational age, mean (y)	39.3 ± 16.8	38.0 ± 1.6	0.352	
Mean BMI (Kg/m^2^) at first prenatal visit	26.8 ± 12.9	29.1 ± 20.2	0.149	
% overweight	32.4 % (121)	31.6 % (36)	0.877	
% obese	21.4 % (80)	27.2 % (31)	0.196	
% exessive weight gain	18.5 % (73)	22.7 % (29)	0.306	
% EMB	21.6 % (97)	33.3 % (48)	**0.004**	0.939
% first degree relative with diabetes	16.7 % (75)	22.9 % (33)	0.107	
% second degree relative with diabetes	23.1 % (104)	17.4 % (25)	0.107	
% history of GDM	10.4 % (47)	21.5 % (31)	**0.002**	0.142
% multiparous	47.6 % (216)	59.3 % (86)	**0.044**	0.998
% primigravida	36.0 % (163)	23.4 % (34)	**0.020**	0.260
Week of GCT, mean	25.4 ± 3.3	23.6 ± 4.9	**<0.0001**	0.203
Value of GCT, mean in mg/dl (mmol/l)	166.7 ± 19.6 (9.3 ± 1.1)	179.0 ± 40.0 (9.9 ± 2.2)	**<0.0001**	0.304
Week of GDM diagnosis mean	27.1 ± 3.7	25.3 ± 4.9	**<0.0001**	0.278
Fasting glycaemia at OGTT in mg/dl (mmol/l)	87.7 ± 10.3 (4.9 ± 0.6)	97.6 ± 18.8 (5.4 ± 1.0)	**<0.0001**	**<0.0001**
1-hour glycaemia at OGTT in mg/dl (mmol/l)	184.5 ± 25.8 (10.2 ± 1.4)	194.7 ± 30.1 (10.8 ± 1.7)	**<0.0001**	0.417
2-hour glycaemia at OGTT in mg/dl (mmol/l)	175.0 ± 22.8 (9.7 ± 1.3)	185.2 ± 28.5 (10.3 ± 1.6)	**<0.0001**	0.342
3-hour glycaemia at OGTT in mg/dl (mmol/l)	145.4 ± 27.6 (8.1 ± 1.5)	152.7 ± 33.4 (8.5 ± 1.8)	**0.013**	0.238
% ≥3 abnormal values on the OGTT	37.8 % (161)	58.1 % (75)	**<0.0001**	0.915
% ≥4 abnormal values at OGTT	7.7 % (33)	24.8 % (32)	**<0.0001**	0.756
HbA1c, mean in % (mmol/mol)	5.2 ± 0.5 (33 ± 6)	5.5 ± 0.6 (37 ± 6)	**<0.0001**	0.496
% treatment with corticoïds	2.4 % (11)	11.0 % (16)	**<0.0001**	

*Y* years, *BMI* body mass index, *EMB* ethnic minority background, *GCT* glucose challenge test, *OGTT* oral glucose tolerance test, *GDM* gestational diabetes *Adjusted *p*-value: Clinical variables that were identified as most significantly associated with the need for insulin use were included in the multivariable logistic regression, P-values in bold are significant values

Table 4 gives an overview of the differences in insulin sensitivity and beta-cell function between the insulin- and diet-treated women with GDM attending the university hospital. In women attending the university hospital, ISSI-2 (1.3 ± 0.5 vs. 1.7 ± 0.5, *p* < 0.0001) and 1/HOMA-IR [0.01 (0.001–0.002) vs. 0.02 (0.01–0.03), *p* = 0.027] were significantly lower in women on insulin, remaining significant after adjustment for age, BMI, excessive weight gain, ethnicity and multi-parity.

**Table 4:** The differences in insulin sensitivity and beta-cell function between the insulin- and diet-treated women with GDM from the university hospital

	Diet *N* = 196(73.0 %)	Insulin *N* = 73(27.0 %)	*P* value	Adjusted *p* value
ISSI-2 index mean	1.7 ± 0.5	1.3 ± 0.5	**<0.0001**	**<0.0001**
Matsuda index median	2.9 (1.9–3.7)	2.3 (1.4–3.3)	**0.019**	0.206
1/HOMA-IR median	0.02 (0.01–0.03)	0.01 (0.01–0.02)	**<0.0001**	**0.027**

GDM: gestational diabetes; ISSI-2: insulin secretion sensitivity index during pregnancy; Matsuda: insulin sensitivity index of Matsuda during pregnancy; 1/HOMA-IR: the reciprocal of the homeostasis model assessment of insulin resistance during pregnancy. The *p*-values are adjusted for age, BMI, excessive weight gain, ethnicity and multi-parity, P-values in bold are significant values

## Discussion

The insulin requirement for the treatment of GDM varies according to the population studied and which screening strategy and diagnostic criteria are used for GDM. Most studies show insulin use in GDM between 10–30 %, in line with the insulin need in our study of 24.1 % [20–22]. Insulin did not prevent adverse outcomes in our study, as women on insulin had higher rates of LGA (28.5 %) and more cesarean sections (44.1 %) compared to diet-treated women (resp. 13.1 %, *p* < 0.0001 and 27.0 %, *p* = 0.001), remaining significant after adjustment for BMI, excessive weight gain, ethnicity, age, multi-parity and center. These data show that especially in women on insulin, rates of LGA and cesarean sections remain much higher compared to the LGA and cesarean rates in pregnant women without GDM of resp. 9.0 % and 23.3 % in our population, as was shown previously by our research group [23]. Controlling hyperglycaemia in pregnancy is certainly not the only essential factor to decrease the problem of LGA newborns. The HAPO study has shown that both maternal GDM and obesity are independently associated with adverse pregnancy outcomes and that the combination of obesity and GDM shows a greater risk of these adverse pregnancy outcomes than either obesity or GDM alone [24]. Gestational weight gain is also a known independent risk factor for accelerated fetal growth [25]. BMI and excessive weight gain were however not different between the two groups in our study but of all women with GDM 32.2 % were overweight and 22.9 % were obese at the first prenatal visit. There is clearly a need for better preconception assessment and counseling for overweight and obese women with the aim to lose weight before pregnancy and also to limit weight gain in pregnancy. In our study oral anti-diabetes drugs were not used during pregnancy. Insulin has been the treatment of choice when lifestyle measures do not maintain glycemic control during pregnancy. The long acting insulins NPH and detemir and the short acting insulins human regular and the insulin analogues lispro and aspart have all been proven to be safe for use during pregnancy [7]. However, insulin is associated with an increased risk for hypoglycaemia and weight gain, which might be a hurdle to reach an optimal glycaemic control and contribute to the increased rates of adverse pregnancy outcomes seen in women with GDM on insulin. Some studies have suggested that metformin may be a safe and acceptable alternative for the treatment of GDM with less maternal weight gain compared to insulin and with no increase in congenital anomalies, despite it crossing the placenta [8, 26, 27]. However there is a paucity of long-term follow up data on children exposed to oral agents in utero. More research is therefore necessary to evaluate whether the addition of metformin to insulin can improve pregnancy outcomes in women with GDM and whether this is also safe on the long term.

Our study also shows that women with GDM on insulin had a more adverse metabolic risk profile compared to diet-treated women with GDM with an earlier diagnosis of GDM, higher glucose values on the OGTT and more often >2 abnormal values on the OGTT. Women on insulin were also more often from an EMB, more often multiparous and more often had a history of GDM. However only the fasting glycaemia at the time of the OGTT was an independent predictor for antenatal insulin requirement in the regression analysis in our population. Other studies have shown independent predictors for antenatal insulin requirement to be a positive family history of diabetes, multiple abnormal values on the OGTT, the BMI, the time of diagnosis, the fasting glucose on the OGTT and HbA1c at the diagnosis of GDM [20–22]. The large variation seen in predictors between the different studies is probably related to the differences in the ethnic origin of the populations studied, the differences in sample size and the different screening strategies used for GDM. Several studies have indicated fasting glucose during the OGTT as a potent predictor of antenatal insulin treatment in GDM, in accordance with our finding [22, 28, 29]. However, depending on the methodology of the study, on the target of glucose levels used or ethnic differences, different cut-off values of fasting glucose varying from 87 mg/dl (4.8 mmol/l) to 105 mg/dl (5.8 mmol/l) have been demonstrated to predict the need for insulin [22, 28, 29]. In our population a FPG cut-off level ≥88.5 mg/dl (4.9 mmol/l) had the best sensitivity and specificity rates but with a low positive predictive value. This FPG cut-off is lower than the general recommended FPG target for the treatment of GDM of < 95 mg/dl (5.3 mmol/l) and is also closer to the FPG cut-off proposed by the IADPSG for the diagnosis of GDM compared to the Carpenter & Coustan criteria we used. This might suggest that a lower FPG target for the treatment of GDM might be indicated but there is a need for large randomized intervention trials evaluating the benefit of lower glycaemic targets for the treatment of GDM on pregnancy outcomes.

Our data also show a reduced insulin sensitivity and impaired beta-cell function in women on insulin, which remained significant after adjustment for confounders such as BMI, age, ethnicity, and excessive weight gain. It has long been thought that the insulin resistance provided a short-term challenge to the beta-cells, with GDM arising in those women whose beta-cells were unable to meet this challenge. Several studies have shown that the defect in beta-cell compensation that characterizes GDM is chronic and probably not just acquired during pregnancy. Shortly after delivery the insulin resistance is generally restored to the pre-pregnancy level but often the chronic beta-cell dysfunction persists [30, 31]. We have previously shown in our population that 39.1 % of women with a recent history of GDM, had glucose intolerance based on the OGTT three months postpartum and that these women also had a persistent impaired beta-cell function and lower insulin sensitivity postpartum [32].

In our cohort, less than one third of women had an abnormal fasting glycaemia on the OGTT while the 2-hour and 3-hour glycaemia values were most frequently abnormal. However, in contrast to the FPG, the 2-and 3-hour glycaemia were not anymore significantly associated with the use of insulin in the multivariable logistic regression. The use of a 2-hour 75 g OGTT with the Carpenter & Coustan criteria for the diagnosis of GDM was accepted as an alternative by the previous ADA recommendations, but this is clearly less validated than the use of the 3-hour 100 g OGTT [1]. If a 2-hour 75 g OGTT would be used in our population with the Carpenter & Coustan criteria for GDM, the prevalence of GDM would decrease importantly since the 3-hour value contributed to the diagnosis of GDM in nearly half of all women with GDM.

Strengths of the study are the detailed characterization of a large cohort of women with GDM using a good database. Variables that were identified as most significantly associated with the need for insulin use were included in a multivariable logistic regression. Pregnancy outcomes and measures of insulin sensitivity and beta-cell function were adjusted for multiple confounders. A limit of the study is the retrospective nature of the analysis and the fact that insulin values were only available from women attending the university hospital.

## Conclusions

Compared to diet-treated women with GDM, women treated with insulin have a higher metabolic risk profile, impaired beta-cell function and lower insulin sensitivity. Moreover, insulin did not prevent adverse outcomes, as rates of LGA and cesarean sections were still higher in insulin-treated women. Women with GDM needing insulin remain a high risk population and more research is necessary to improve outcomes in this group.
